# Analysis of clinical characteristics of thyroid disorders in patients with chronic hepatitis B treated with pegylated-interferon alpha

**DOI:** 10.1186/s12902-023-01371-w

**Published:** 2023-05-22

**Authors:** Yisi Liu, Yanhong Zheng, Xiao Lin, Zhenhuan Cao, Junfeng Lu, Lina Ma, Shan Ren, Sujun Zheng, Zhongjie Hu, Bin Xu, Xinyue Chen

**Affiliations:** 1 First Department of Liver Disease Center, Beijing Youan Hospital, Capital Medical University, No.8, Xi Tou Tiao, Youanmen Wai, Beijing, 100069 Fengtai District China; 2 Third Department of Liver Disease Center, Beijing Youan Hospital, Capital Medical University, Beijing, China; 3 Second Department of Liver Disease Center, Beijing Youan Hospital, Capital Medical University, Beijing, China

**Keywords:** Thyroid diseases, Chronic hepatitis B, Interferon alpha, Side effect, Functional cure

## Abstract

**Background:**

Thyroid disorders (TD) is a common complication of pegylated-interferon alpha (Peg-IFNα) therapy. Few studies have investigated the relationship between TD and the efficacy of interferon therapy for chronic hepatitis B (CHB). Therefore, we analyzed the clinical characteristics of TD in patients with CHB treated with Peg-IFNα, and evaluated the correlation between TD and Peg-IFNα treatment efficacy.

**Methods:**

In this retrospective study, the clinical data of 146 patients with CHB receiving Peg-IFNα therapy were collected and analyzed.

**Results:**

During the course of Peg-IFNα therapy, positive conversion of thyroid autoantibodies and TD occurred in 7.3% (85/1158) and 8.8% (105/1187) patients, respectively, and was diagnosed more often in women. The most common thyroid disorder was hyperthyroidism (53.3%), followed by subclinical hypothyroidism (34.3%). We found that thyroid function returned to normal in 78.7% of patients with CHB, and thyroid antibody levels returned to the negative range in approximately 50% of patients after interferon treatment cessation. Only 25% of patients with clinical TD required treatment. Compared with patients with hypothyroidism/subclinical hypothyroidism, patients with hyperthyroidism/subclinical hyperthyroidism showed greater reduction and seroclearance of hepatitis B surface antigen (HBsAg) levels.

**Conclusions:**

TD are not an absolute contraindication for interferon therapy; however, patients should be monitored closely during interferon therapy. In pursuit of functional cure, a balance between efficacy and safety must be achieved.

**Supplementary Information:**

The online version contains supplementary material available at 10.1186/s12902-023-01371-w.

## Background

Chronic hepatitis B virus (HBV) infection is a serious public health problem and is the main cause of liver cirrhosis and hepatocellular carcinoma (HCC) [1]. The functional cure for chronic hepatitis B (CHB), which include sustained hepatitis B surface antigen (HBsAg) seroclearance after drug withdrawal, with or without HBsAg seroconversion, is the most ideal therapeutic goal recommended by the current guidelines, and is closely related to reduction of liver cirrhosis and HCC [2–4]. Currently, antiviral drugs for CHB mainly include nucleos(t)ide analogs (NA) and pegylated-interferon alpha (Peg-IFNα). The seroclearance of HBsAg treated with NA alone is incredibly low, which is close to 1% of the natural seroclearance rate [5]. Recently, interferon-based optimal treatment strategies have significantly increased the probability of functional cure (14.4–44.7%) [6, 7]. However, the adverse reactions of interferon limit its wide use in clinical setting. At present, clinicians can skillfully deal with some common side effects of interferon alpha (IFNα) such as flu-like symptoms and hemocytopenia; however, thyroid disorders (TD) has become a major problem restricting its clinical use. In the past, IFNα can cause TD during the treatment of chronic hepatitis C (CHC), and the sustained virological response rate of patients with TD is considerably higher than that of those without TD (84.2% vs. 53.9%, *p* < 0.05) [8–10]. Despite its clinical implications, the incidence of TD during IFNα-based treatment strategies for CHB, and how it relates to efficacy are rarely reported. Thus, we retrospectively analyzed the clinical characteristics of TD during the course of interferon therapy and assessed the relationship between TD occurrence and interferon therapy efficacy to provide more data for balance safety and efficacy in pursuit of functional cure for CHB.

## Materials and methods

### Study participants

**Fig. 1:**
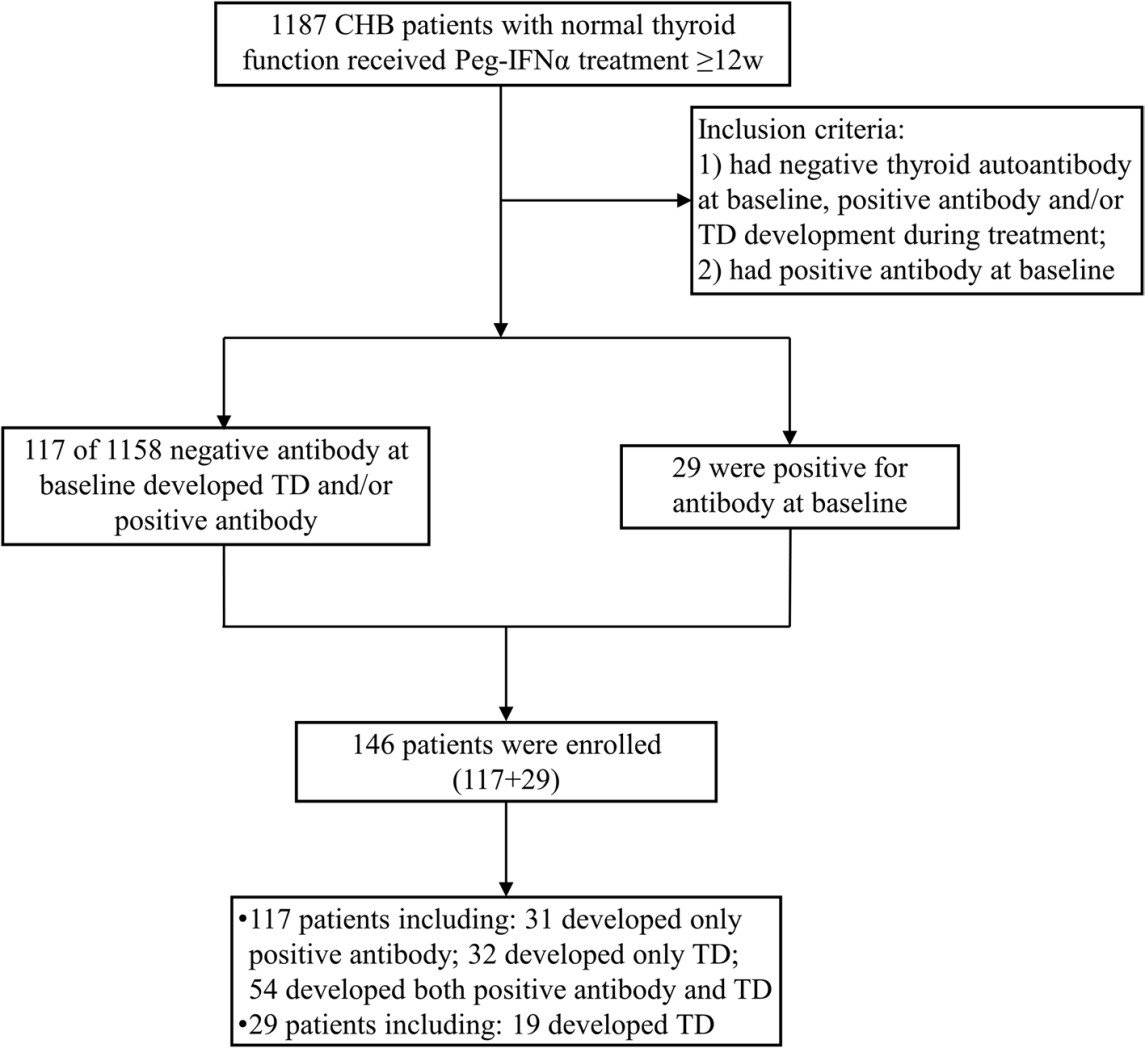
Screening population and enrollment situation

The participants of this study included patients with CHB who visited the Beijing Youan Hospital, affiliated with Capital Medical University from January 2017 to June 2021. We used the diagnostic criteria for CHB ‘The guideline of prevention and treatment for chronic hepatitis B: a 2015 update’ [11]. The screening population had normal thyroid function before treatment and had been treated with Peg-IFNα for at least 12 weeks. Inclusion criteria: 1) had negative thyroid autoantibody (hereinafter referred to as antibody) at baseline, positive antibody and/or TD development during treatment; 2) had positive antibody at baseline (Fig. 1). This study was approved by the research ethics committee of the Beijing Youan Hospital, Capital Medical University, China ([2022]046).

### Study design

A retrospective study design was applied, and demographic and clinically relevant data were obtained from patient medical records. Laboratory data including HBV infection markers, biochemical indicators, thyroid function indicators, and autoantibody levels. All patients receiving Peg-IFNα were measured the above indicators before Peg-IFNα treatment, every 12 weeks during the course of treatment, and 24 weeks after treatment cessation.

### Detection of clinical indexes

HBV serological markers were measured by Elecsys immunoassay (Roche, Basel, Switzerland) with a lower detection limit for HBsAg of 0.05 IU/mL and 1 COI for hepatitis B e antigen (HBeAg). Thyroid autoantibodies were detected using a Roche Cobas e801 module. Negative results were defined as: thyroglobulin antibody (TGAb) < 115 IU/mL; thyroid peroxidase antibody (TPOAb) < 34 IU/mL; and thyrotropin receptor antibody (TRAb) < 1.75 IU/mL. Thyroid function was assessed using an Architect i2000 immunoassay analyzer (Abbott Laboratories, Abbott Park, IL, USA). The reference ranges for thyroid function indicators were: free triiodothyronine, 3.6–6.5 pmol/L; triiodothyronine, 0.92–2.79 nmol/L; free thyroxine, 11.5–22.7 pmol/L; thyroxine, 58.1–140.6 nmol/L; and thyroid stimulating hormone, 0.55–4.78 mIU/L.

### Definition of disease

Functional cure of hepatitis B was defined as HBsAg of < 0.05 IU/mL, negative HBeAg, HBV DNA below the lower detection limit, and normal alanine transaminase.

The definition of abnormal thyroid function in this study was mainly based on the guidelines of the American Thyroid Association as follows [12]: hyperthyroidism was defined as a low or undetectable serum TSH with elevated serum levels of T3, FT3, TT4, and/or FT4; subclinical hyperthyroidism was defined as a low or undetectable serum TSH with values within the normal reference range for TT3, FT3 TT4, and FT4; hypothyroidism was defined as a high serum TSH with decreased serum levels of T3, FT3, TT4, and/or FT4; and subclinical hypothyroidism was defined as a high serum TSH with values within the normal reference range for TT3, FT3 TT4, and FT4.

Additionally, abnormal thyroid function was generally classified as TD in this study.

### Statistical analysis

SPSS version 22.0 software (IBM Corp., Armonk, NY, USA) was used for data processing and statistical analysis. Approximately normally distributed data are expressed as the mean ± standard deviation. The Mann–Whitney test was used to compare measurement data with skewed distribution between groups. Categorical variables were analyzed using χ2 or Fisher's exact tests. Multivariate logistic regression was used for the analysis of influencing factors associated with antiviral efficacy; *p* < 0.05 indicated a statistically significant difference.

## Results

### General condition of patients

A total of 1187 CHB patients with normal thyroid function were voluntarily treated with Peg-IFNα between January 2017 and June 2021; of which, 146 met the inclusion criteria (Fig. 1). The treatment regimen used for all enrolled patients was a combination of NA and Peg-IFNα (119 patients received entecavir, 23 patients received tenofovir disoproxil fumarate, and 4 patients received tenofovir alafenamide fumarate; the Peg-IFNα dose was 135–180 μg/week). There were 58 men and 88 women, with an average age of 37.09 years; the average age of women was younger than that of men (*p* = 0.007). The mean HBsAg level at baseline was 2.87 ± 1.13 logIU/mL, and there was no statistical difference with respect to sex. There were 62 CHB patients positive for HBeAg. Before Peg-IFNα treatment, 29 patients were positive for antibodies, whereas 117 patients were negative (Table 1). Among the 29 patients with antibody positivity, 11 were positive only for TGAb, 14 were positive for only TPOAb, 3 were positive for both TGAb and TPOAb, and 1 was positive for TGAb, TPOAb, and TRAb.

**Table 1:** General condition of 146 patients

	total (*n* = 146)	male (*n* = 58)	female (*n* = 88)	*p* value
Age (year)	37.09 ± 8.51	39.41 ± 8.65	35.56 ± 8.11	0.007
Median course of treatment (weeks)	36	48	36	0.004
range	12–96			
Baseline HBsAg level (logIU/ml)	2.87 ± 1.13	2.67 ± 1.12	3 ± 1.12	0.084
HBeAg positive (%)	62 (42.5%)	19 (32.8%)	43 (48.9%)	0.054
Baseline ALT (U/L)	20 (14, 37)	28.5 (17, 42.75)	18 (13, 30.5)	0.009
Baseline thyroid autoantibody positive	29	12	17	

*ALT* aspartate aminotransferase, *HBsAg* hepatitis B surface antigen

### Occurrence of antibody positivity and thyroid disorder during the course of treatment

TD occurred in 19 of 29 patients with CHB and antibody positivity at baseline who were treated with PEG-IFNα. Among the remaining 1158 patients with antibody negativity at baseline, 117 patients (10.1%) developed positive conversion of antibodies or TD during Peg-IFNα treatment. Among these 117 patients, 31 (2.7%) patients developed only positive conversion of antibodies, 32 (2.7%) patients developed only TD, and 54 (4.7%) patients developed both positive conversion of antibodies and TD (Fig. 1).

A total of 85 (31 + 54; 7.3%) patients experienced positive conversion of antibodies during Peg-IFNα treatment. The positive conversion rate of antibodies in women was 11.4%, which was significantly higher than that in men (4.4%) (*p* < 0.001). In addition, the positive conversion rates of TGAb, TPOAb, and TRAb antibodies in women were all significantly higher than those in men (*p* < 0.05). The median time to positive conversion of antibodies was 24 weeks for women and 36 weeks for men, and the difference was statistically significant (*p* = 0.037) (Table 2). The changes of thyroid function and thyroid autoantibodies before and after interferon treatment are detailed in Supplementary Table 1.

**Table 2:** Occurrence and recovery of thyroid autoantibodies

	total (*n* = 1158)	male (*n* = 666)	female (*n* = 492)	*p* value
Antibodies positive rate during treatment	7.3% (85/1158)	4.4% (29/666)	11.4% (56/492)	0
TGAb	4.2% (49/1158)	1.7% (11/666)	7.7% (38/492)	0
TPOAb	4.4% (51/1158)	2.7% (18/666)	6.7% (33/492)	0.001
TRAb	2.3% (27/1158)	1.1% (7/666)	4.1% (20/492)	0.001
Median time to antibody positive (weeks)	24	36	24	0.037
Negative conversion rates of antibody in 24 weeks				
TGAb	52.1% (25/48)	81.8% (9/11)	43.2% (16/37)	0.025
TPOAb	30.6% (15/49)	25% (4/16)	33.3% (11/33)	0.553
TRAb	53.6% (15/28)	62.5% (5/8)	50% (10/20)	0.857

*TGAb* thyroglobulin antibody, *TPOAb* thyroid peroxidase antibody, *TRAb* thyrotropin receptor antibody

A total of 8.8% (105/1187) patients developed TD during the treatment period. Of which 19 were positive for thyroid antibodies at baseline; the remaining 86 (32 + 54) patients were negative for thyroid antibodies at baseline. The incidence of TD in women (12.8%) was significantly higher than that in men (5.9%) (*p* < 0.001). The median time of the occurrence of TD was 18 weeks in patients with antibody positivity at baseline, which was significantly shorter than that in patients with antibody negativity at baseline (24 weeks) (*p* = 0.009). In terms of differences in the time of TD after interferon treatment between sexes, although the median time was 24 weeks, the average rank of women was significantly lower than that of men (*p* = 0.024). The analysis of the clinical types of TD revealed that hyperthyroidism accounted for 53.3%, subclinical hypothyroidism for 34.3%, and subclinical hyperthyroidism and hypothyroidism for 5.7% and 6.7%, respectively. No significant difference was observed in the clinical types of TD between sexes. Among patients with clinical TD, 25% had clinical symptoms and 12.7% received antithyroid drugs or replacement therapy (Table 3). Among the 56 patients with hyperthyroidism, 18 were positive for TRAb, 25 were negative for TRAb but positive for TGAb and/or TPOAb, and 13 were negative for antibodies.

**Table 3:** Occurrence and recovery of thyroid diseases

	total (*n* = 1187)	male (*n* = 678)	female (*n* = 509)	*p* value
Rate of TD during treatment	8.8% (105/1187)	5.9% (40/678)	12.8% (65/509)	0
hyperthyroidism	53.3% (56/105)	45% (18/40)	58.5% (38/65)	0.525
subclinical hyperthyroidism	5.7% (6/105)	5% (2/40)	6.2% (4/65)	
hypothyroidism	6.7% (7/105)	7.5% (3/40)	6.2% (4/65)	
subclinical hypothyroidism	34.3% (36/105)	42.5% (17/40)	29.2% (19/65)	
Median time to TD (weeks)	24	24	24	0.024
Baseline antibody positive	18			0.009
Baseline antibody negative	24			
Symptom ratio of clinical TD	25% (14/56)	15% (3/20)	30.6% (11/36)	0.198
Rate of receiving antithyroid drugs or replacement therapy	12.7% (7/55)	15.8% (3/19)	11% (4/36)	0.621
normalization rate of TD at 24 weeks after discontinue treatment	78.7% (74/94)	84.8% (28/33)	75.4% (46/61)	0.286
Hyperthyroidism/subclinical hyperthyroidism	74.5% (41/55)			0.24
Hypothyroidism/subclinical hypothyroidism	84.6% (33/39)			

*TD* thyroid disorders

### Recovery of antibody levels and thyroid function after treatment

At the 24-week time point after Peg-IFNα treatment cessation, the negative conversion rates of TGAb and TRAb were 52.1% and 53.6%, respectively, although the negative conversion rate of TPOAb was less than one third (30.6%). The negative conversion rate of TGAb in women was significantly lower than that of men (43.2% vs 81.8%) (*p* = 0.025) (Table 2). The normalization rate of TD was 78.7%, and the normalization rates of hyperthyroidism/subclinical hyperthyroidism and hypothyroidism/subclinical hypothyroidism were 74.5% and 84.6%, respectively (Table 3). We further followed-up patients whose thyroid function did not return to normal at the end of 24–72 weeks. We found that only 2 of the 14 patients with hyperthyroidism/subclinical hyperthyroidism whose thyroid function did not return to normal; however, these 2 patients with hyperthyroidism reverted to subclinical hypothyroidism. Meanwhile, the thyroid function of only 1 of the 6 patients with hypothyroidism/subclinical did not return to normal.

### Relationship between thyroid disorders and curative effect

In this study, two time points, that is 24 and 48 weeks of treatment, were chosen. The HBsAg seroclearance rate, decreased HBsAg levels by more than 1log from baseline, and HBeAg seroclearance rate were used as efficacy evaluation indicators to analyze the correlation between TD and curative effect. After 24 weeks of treatment, the proportion of patients with hyperthyroidism/subclinical hyperthyroidism with HBsAg levels that decreased by more than 1log was 40.7%, which was significantly higher than that in patients with hypothyroidism/subclinical hypothyroidism (*p* = 0.007). The HBsAg seroclearance rate was as high as 37.9% in patients with hyperthyroidism/subclinical hyperthyroidism and only 5.7% in patients with hypothyroidism/subclinical hypothyroidism after 48 weeks of treatment (*p* = 0.001) (Table 4). In the subgroup analysis of hyperthyroidism, participants were divided into two groups according to whether they were TRAb positive or not, and no correlation with efficacy was found (Supplementary Table 2). Additionally, no difference was found between the abnormal thyroid and normal thyroid function groups regarding HBsAg decline > 1 log and HBsAg seroclearance at 24 and 48 weeks (Supplementary Table 3).

**Table 4:** The relationship between TD and efficacy of Peg-IFNα

	Hyperthyroidism/subclinical hyperthyroidism	Hypothyroidism/subclinical hypothyroidism	*p* value
HBsAg seroclearance rate in 24 weeks	12.9% (8/62)	7% (3/43)	0.525
Rate of decreasing in HBsAg levels > 1log in 24 weeks	40.7% (22/54)	15% (6/40)	0.007
HBeAg seroclearance rate in 24 weeks	21.7% (5/23)	31.6% (6/19)	0.712
HBsAg seroclearance rate in 48 weeks	37.9% (11/29)	5.7% (2/35)	0.001
Rate of decreasing in HBsAg levels > 1log in 48 weeks	52.6% (10/19)	27.2% (9/33)	0.067
HBeAg seroclearance rate in 48 weeks	55.5% (5/9)	47.4% (9/19)	1

*TD* thyroid disorders, *Peg-IFNα* pegylated-interferon alpha, *HBsAg* hepatitis B surface antigen

### Regression analysis of factors affecting the decline of HBsAg level

**Fig. 2:**
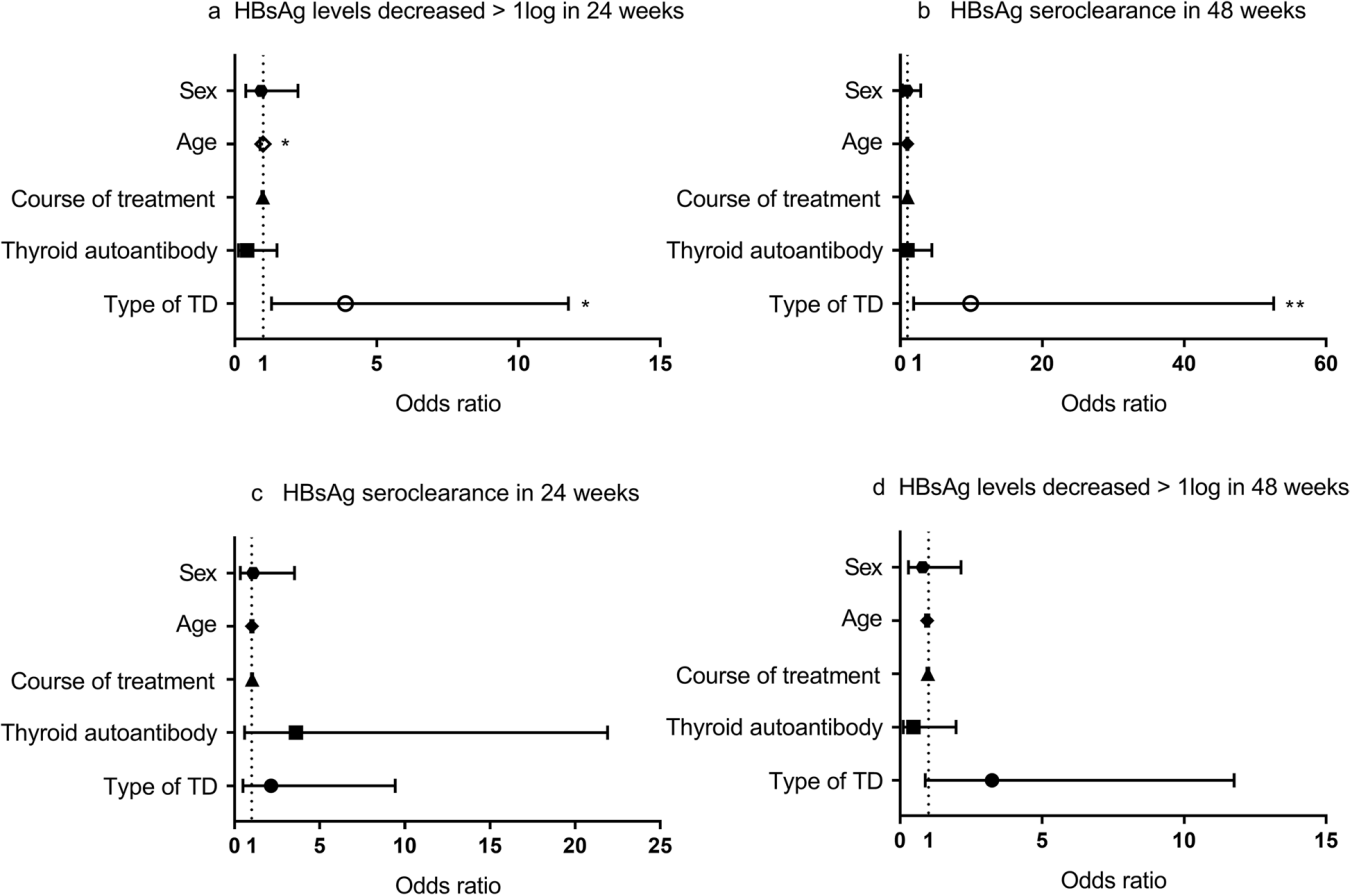
Factors affecting the efficacy of Peg-IFNα. The OR and 95% CI was plotted for each variable included in the multivariate logistic regression models (sex, female/male; thyroid autoantibody, positive/negative; TD type, clinical or subclinical hyperthyroidism/clinical or subclinical hypothyroidism). CI, confidence interval; OR, odds ratio; Peg-IFNα, pegylated-interferon alpha; TD, thyroid disorder; *p < 0.05; **p < 0.01

The HBsAg seroclearance and levels that decreased by more than 1log from baseline after 24 and 48 weeks of treatment were used as the dependent variables. Sex, age, course of Peg-IFNα treatment, thyroid autoantibody positivity, and type of TD were entered as independent variables into the logistic regression model. The results showed that after 24 weeks of treatment, patients with hyperthyroidism/subclinical hyperthyroidism had 2.9-times higher odds of exhibiting a decrease in HBsAg levels of more than 1log compared with patients with hypothyroidism/subclinical hypothyroidism (OR = 3.906, *p* = 0.015) (Fig. 2a). In terms of the HBsAg seroclearance rate after 48 weeks of treatment, patients with hyperthyroidism/subclinical hyperthyroidism had 8.9-times higher odds of HBsAg seroclearance compared with patients with hypothyroidism/subclinical hypothyroidism (OR = 9.901, *p* = 0.007) (Fig. 2b). For every 1-year increase in age, the odds of a 1-log decrease in HBsAg level decreased by 5.6% after 24 weeks of treatment (OR = 0.994, *p* = 0.042) (Fig. 1A). However, there was no significant difference in the above independent variables in the aspect of HBsAg seroclearance after 24 weeks and HBsAg levels that decrease by more than 1log after 48 weeks of treatment (*p* > 0.05) (Fig. 2c/2d).

## Discussion

A retrospective study by Kozielewicz et al. reported a 7.1% (7/99) probability of occurrence of TD in patients with CHB receiving Peg-IFNα treatment [13]. Our results were similar, with approximately 8.8% (105/1187) of patients developing TD during treatment. The mechanism by which interferon causes TD remains unclear, although it is certain that the immune system plays an essential role. Interferon binding to the receptor activates the JAK-STAT pathway, resulting in the transcription of a large number of interferon-stimulated genes, thereby inducing autoimmune thyroiditis [14]. Prummel et al. pooled the results of multiple studies and found that TPOAb positivity before treatment was associated with a 3.9-fold increased risk of TD [15]. Koh et al. analyzed the clinical data of interferon therapy in the treatment of tumors and immune diseases and uncovered that the incidence of TD was higher in patients with thyroid antibody positivity before treatment (46.1%, 41/89) [16]. In our study, patients with baseline antibody-positive CHB were found to have a higher TD incidence (65.5%, 9/29) after interferon treatment, and the time to occurrence of TD was found to be significantly shorter in patients with antibody positivity at baseline compared with patients with antibody negativity (the median time was 18 and 24 weeks, respectively). Moreover, this study also suggested that as Peg-IFNα treatment continued, the positive conversion rate of antibodies increased significantly, from 2.4% (29/1187) at baseline to 9.6% (114/1187). Specifically, the overall positive conversion rate of thyroid autoantibody and the individual positive conversion rates of TGAb, TPOAb, and TRAb in women were all significantly higher than those in men. Meanwhile, the TD rate of women was also higher, although the negative conversion rate of TGAb in women was significantly lower than that in men. A similar study reported that when interferon was used to treat diseases other than CHB, women had a 4.4-times greater risk of TD than that of men [15]. Therefore, the administration of Peg-IFNα should be monitored more closely in patients with thyroid antibody positivity, especially among women. However, not all TD were associated with thyroid antibodies. We found that 30.5% (32/105) of patients only presented with TD and antibody negativity. It has been speculated that the pathogenesis of this type of TD is different from that described above, and some studies have suggested that genetic factors play a role. Dalgard et al. reported that among patients with CHC treated with interferon, the incidence of TD was significantly higher in Asian populations compared with other ethnic groups [17]. Some studies have also found that HLA-A2 is related to Peg-IFNα-induced autoimmune thyroid diseases [18]. In addition, the direct toxic effect of interferon on thyroid cells can also lead to thyroid diseases [19, 20].

Regarding the types of TD caused by interferon, this study indicated that hyperthyroidism accounted for 53.3%, followed by subclinical hypothyroidism (34.3%). Okanoue et al. studied 987 patients with CHC receiving short-acting interferon and found that 12 of 18 patients who developed TD had hyperthyroidism and 6 had hypothyroidism [21]. In another study that included 422 patients with chronic hepatitis C, B, and D infections, the main type of TD caused by IFNα was hypothyroidism, accounting for 83.3% (25/30) of cases, while only 5 patients had hyperthyroidism [22]. The different proportions of TD types among studies may be related to different types of diseases and interferons used. Further analysis of the correlation between the clinical types of TD and curative effect revealed that the proportion of those with > 1log reduction in HBsAg levels from baseline after 24 weeks of treatment and HBsAg seroclearance after 48 weeks of treatment were both significantly higher in patients with CHB with hyperthyroidism/subclinical hyperthyroidism than in patients with hypothyroidism/subclinical hypothyroidism. Furthermore, logistic regression analysis suggested that the odds of HBsAg levels decreasing by more than 1log from baseline after 24 weeks and HBsAg seroclearance after 48 weeks was 3.9- and 9.9-fold, respectively, in patients with hyperthyroidism/subclinical hyperthyroidism compared with patients with hypothyroidism/subclinical hypothyroidism. In the subgroup analysis of hyperthyroidism, there was no significant difference in the HBsAg decline between TRAb positive and negative patients with hyperthyroidism. Reports on the correlation between TD and clinical efficacy of interferon treatment are limited, save for one study by Luo et al. [23]. The authors observed positive conversion of antibodies or TD in 141 of 342 patients with CHB treated with Peg-IFNα, and there was a more pronounced decrease in HBsAg levels in these patients (*p* = 0.019). However, the study did not categorize TD into specific clinical types. In our study, most TD patients had a good prognosis, and the thyroid function in 78.7% of the patients returned to normal after discontinuing Peg-IFNα for 6 months. Approximately 50% of patients had negative conversion of thyroid antibodies, and antithyroid drugs or thyroid hormone replacement therapy was only required in 25% of patients with clinical TD. Studies on IFNα therapy in CHC patients have also revealed a high normalization rate of TD after discontinuation of interferon therapy, with a normalization rate of 87.5% in 24 patients approximately 1.5 years after discontinuation [24]. Based on the pooled analysis of multiple studies, Mandac et al. believe that interferon therapy can be continued in the context of symptomatic treatment and close monitoring [9]. However, in patients with TD with clinical symptoms, discontinuation of interferon therapy and consultation with an endocrinologist should be considered. According to the relevant guidelines in the United States, β-blockers and other symptomatic treatments should be administered to patients with clinical hyperthyroidism during the course of interferon therapy, and antithyroid drugs and radiation therapy should only be used in patients with Graves' disease [12].

A study has found that adding thyroid hormone to human fibroblasts cultured in vitro increases the level of interferon gamma, and inhibits the proliferation of vesicular stomatitis virus [25]. Similar phenomena were also observed in clinical studies. The study found that the levels of interleukin-6 (IL-6), tumor necrosis factor-α (TNF-α), and chemokines were significantly increased in IFNα-treated CHC patients with TD, and these cytokines have immune function-enhancing effects [26]. In another case–control study, sustained virological response rates were higher in CHC patients who develop TD with IFNα therapy, and 94.7% (18/19) of the TD types were hyperthyroidism [10]. This is the double-edged sword effect of immune upregulation and immune damage during interferon therapy. Our study showed that the transaminase elevation in the early stage (12 weeks) of Peg-IFNα treatment for CHB could serve as a strong predictor of HBsAg seroclearance [7]. However, the up-regulated immune response may not be precise. Immune clearance is necessary for the treatment of CHB, although it will inevitably result in immune damage to the thyroid and other self-organized tissues. This requires clinicians to consider maximizing the interests of patients when dealing with TD and make an individualized treatment plan. For asymptomatic patients with hyperthyroidism, a reduced dose of Peg-IFNα should be considered if the HBsAg has reached relatively low levels to warrant the continued pursuit of HBsAg seroclearance. For patients with unsatisfactory decline in HBsAg levels and hyperthyroidism, discontinuation of treatment should be considered. In antiviral therapy for CHB, virological and serological response should be pursued, and the safety of treatment should also be considered. For patients who are in the process of receiving Peg-IFNα treatment, thyroid function and related antibody detection should be performed before treatment to assess the TD risk, and regular review is needed during the course of treatment to early detect TD and respond accordingly.

In conclusion, this study suggests that a certain proportion of positive conversion of thyroid antibodies (7.3%) and TD (8.8%) will occur during the course of Peg-IFNα treatment for CHB and is more common in women. Overall, the clinical symptoms of TD were mild; treatment was only needed in 25% of patients, and the normalization rate of TD reached 78.7%. Compared with patients with hypothyroidism/subclinical hypothyroidism, patients with hyperthyroidism/hypothyroidism have higher odds of reduced HBsAg levels and HBsAg seroclearance. Therefore, we believe that TD is not an absolute contraindication for Peg-IFNα therapy. In the course of treatment, patients should be monitored closely to balance efficacy and safety in the pursuit of functional cure.

## Supplementary Information

**Additional file 1:** **Supplementary Table 1. **Thyroid function and antibody changes before and after treatment.** Supplementary Table 2. **Subgroup analysis of hyperthyroidism.** Supplementary Table 3. **Comparison between abnormal thyroid function group and nomal group.

## Data Availability

The data that support the findings of this study are available from the corresponding author upon reasonable request.

## Abbreviations

ALT: Aspartate aminotransferase
CHB: Chronic hepatitis B
CHC: Chronic hepatitis C
CI: Confidence interval
HBsAg: Hepatitis B surface antigen
HCC: Hepatocellular carcinoma
HBV: Chronic hepatitis B virus
interferon alpha: IFNα
NA: Nucleos(t)ide analogs
OR: Odds ratio
Peg-IFNα: Pegylated-interferon alpha
TD: Thyroid disorders
TGA: Thyroglobulin antibody
TPOAb: Thyroid peroxidase antibody
TRAb: Thyrotropin receptor antibody
