# Supplementary material for: Analysis of clinical characteristics of thyroid disorders in patients with chronic hepatitis B treated with pegylated-interferon alpha
Source: BMC Endocr Disord. 2023 May 22;23:115. doi: 10.1186/s12902-023-01371-w (PMC10201755; doi:10.1186/s12902-023-01371-w)
Supplement: Supplementary file 1 — Additional file 1: Supplementary Table 1. Thyroid function and antibody changes before and after treatment. Supplementary Table 2. Subgroup analysis of hyperthyroidism. Supplementary Table 3. Comparison between abnormal thyroid function group and nomal group. [file 12902_2023_1371_MOESM1_ESM.docx]

**Supplementary Table 1.** Thyroid function and antibody changes before and after treatment

|  | Baseline | Level at discontinuation | p value |
| --- | --- | --- | --- |
| FT3, pmol/L | 4.68±0.62 | 6.07±3.84 | <0.001 |
| TT3, nmol/L | 1.63±0.25 | 2.29±0.96 | <0.001 |
| FT4, pmol/L | 13.85±2.07 | 15.88±7.12 | <0.001 |
| TT4, nmol/L | 100.39±20.95 | 126.46±49.71 | <0.001 |
| TSH, mIU/L | 1.98±1.85 | 3.21±4.83 | <0.001 |
| TGAb, IU/mL | 10.9 | 110.3 | <0.001 |
| TPOAb, IU/mL | 11.85 | 45.37 | <0.001 |
| TRAb, IU/mL | 0.3 | 0.8 | NA |

FT3, free triiodothyronine; TT3, triiodothyronine; FT4, free thyroxine; TT4, thyroxine; TSH, thyroid stimulating hormone; TGAb, thyroglobulin antibody; TPOAb, thyroid peroxidase antibody; TRAb, thyrotropin receptor antibody

**Supplementary Table 2.** Subgroup analysis of hyperthyroidism

|  | TRAb positive  N=18 | TRAb negative  N=38 | *p* value |
| --- | --- | --- | --- |
| HBsAg seroclearance rate in 24 weeks | 22.22% (4/18) | 10.52% (4/38) | 0.448 |
| Rate of decreasing in HBsAg levels > 1log in 24 weeks | 50% (7/14) | 38.23% (13/34) | 0.452 |
| HBeAg seroclearance rate in 24 weeks | 40% (2/5) | 18.75% (3/16) | 0.33 |
| HBsAg seroclearance rate in 48 weeks | 37.5% (3/8) | 33.33% (6/18) | 1 |
| Rate of decreasing in HBsAg levels > 1log in 48 weeks | 80% (4/5) | 46.15% (6/13) | 0.444 |
| HBeAg seroclearance rate in 48 weeks | 100% (3/3) | 33.33% (2/6) | 0.167 |

TRAb, thyrotropin receptor antibody

|  | Thyroid dysfuncion group  N=105 | Normal thyroid function group  N=41 | *p* value |
| --- | --- | --- | --- |
| HBsAg seroclearance rate in 24 weeks | 10.48% (11/105) | 9.76% (4/41) | 1 |
| Rate of decreasing in HBsAg levels > 1log in 24 weeks | 29.79% (28/94) | 21.62% (8/37) | 0.346 |
| HBeAg seroclearance rate in 24 weeks | 26.19% (11/42) | 15% (3/20) | 0.509 |
| HBsAg seroclearance rate in 48 weeks | 20.31% (13/64) | 14.71% (5/34) | 0.495 |
| Rate of decreasing in HBsAg levels > 1log in 48 weeks | 36.54% (19/52) | 34.48% (10/29) | 0.853 |
| HBeAg seroclearance rate in 48 weeks | 50% (14/28) | 18.75% (3/16) | 0.041 |

**Supplementary Table 3.** Comparison between abnormal thyroid function group and nomal group
